# Baicalin alleviates intestinal inflammation and microbial disturbances by regulating Th17/Treg balance and enhancing *Lactobacillus* colonization in piglets

**DOI:** 10.1186/s40104-024-01126-0

**Published:** 2024-12-20

**Authors:** Shunfen Zhang, Chengzeng Luo, Kai Li, Junhong Wang, Huixin Wang, Ruqing Zhong, Liang Chen, Qiugang Ma, Hongfu Zhang

**Affiliations:** 1 State Key Laboratory of Animal Nutrition and Feeding, Institute of Animal Science, Chinese Academy of Agricultural Sciences, Beijing, 100193 China; 2 College of Animal Science and Technology, China Agricultural University, Beijing, 100193 China

**Keywords:** Baicalin, *Escherichia coli*, Intestinal inflammation, Microbiota, Piglets, Th17/Treg balance, Th17 cell

## Abstract

**Background:**

Intestinal inflammation is a common and serious health problem in piglet production, especially enteritis caused by pathogenic *Escherichia coli* (*E. coli*). This condition often leads to high mortality, slow weight gain, and significant economic losses.

**Results:**

In this study, we isolated an *E. coli* strain, SKLAN202302, from the colon of diarrheal piglets to create an intestinal inflammation model for evaluating the protective effects of baicalin. Piglets infected with *E. coli* exhibited significant reductions in body weight, feed intake, small intestine length, and ileal goblet cell count (*P* < 0.05), along with deteriorated ileal morphology. However, baicalin supplementation resulted in body weights, feed intake, and intestinal morphology similar to those of the control group. Notably, there was a significant increase in the colonization of *Lactobacillus* species, particularly *Lactobacillus_reuteri*, *Lactobacillus_amylovorus*, and *Lactobacillus_johnii*, compared to the *E. coli* group (*P* < 0.05). At the metabolic and transcriptional levels, *E. coli* infection increased inflammatory mediators, including eicosanoids (leukotriene F4, prostaglandin F1a, leukotriene E4, thromboxane B2, prostaglandin G2, and PGH2), monosaccharides, and TCA cycle intermediates (oxoglutaric acid, glutaric acid, adipic acid, citric acid, and isocitric acid) in the ileum. It also promoted the expression of genes related to autoimmune diseases and the Th17 differentiation signaling pathway (*CTLA4*, *IFN-ALPHA-8*, *IL12RB2*, *TRAV3*, *TRAV16*, *FOS*, and *VEGFA*), as well as inflammatory factors. Conversely, baicalin supplementation not only counteracted these effects but also enhanced the presence of metabolites such as phospholipids [including lysoPC (P-18:1(9Z)/0:0), PC (17:0/0:0), lysoPC (16:1(9Z)/0:0), PC (18:0/0:0), lysoPC (18:0/0:0), PA (10:0/i-16:0), and PA (10:0/8:0)] and amino acids. It also regulated genes within the IL-17 signaling pathway (*IL4*, *CCL17*, *CXCL10*, *IFNG*, and *CXCL2*), suggesting a mechanism by which baicalin mitigates *E. coli*-induced intestinal and microbial disturbances. Subsequent flow cytometry analysis showed that *E. coli* infection increased the numbers of CD3^+^ and Foxp3^+^ cells, decreased IL-17A^+^ cells, and reduced Th17/Treg ratios. Baicalin supplementation restored these parameters to control levels.

**Conclusions:**

Baicalin supplementation effectively alleviates *E. coli*-induced intestinal inflammation and microbial disturbances in piglets by enhancing beneficial *Lactobacillus* colonization, counteracting inflammatory mediators, and regulating immune-related gene expression and the Th17/Treg balance. These findings highlight baicalin’s potential in alleviating intestinal inflammation.

**Graphical abstract:**

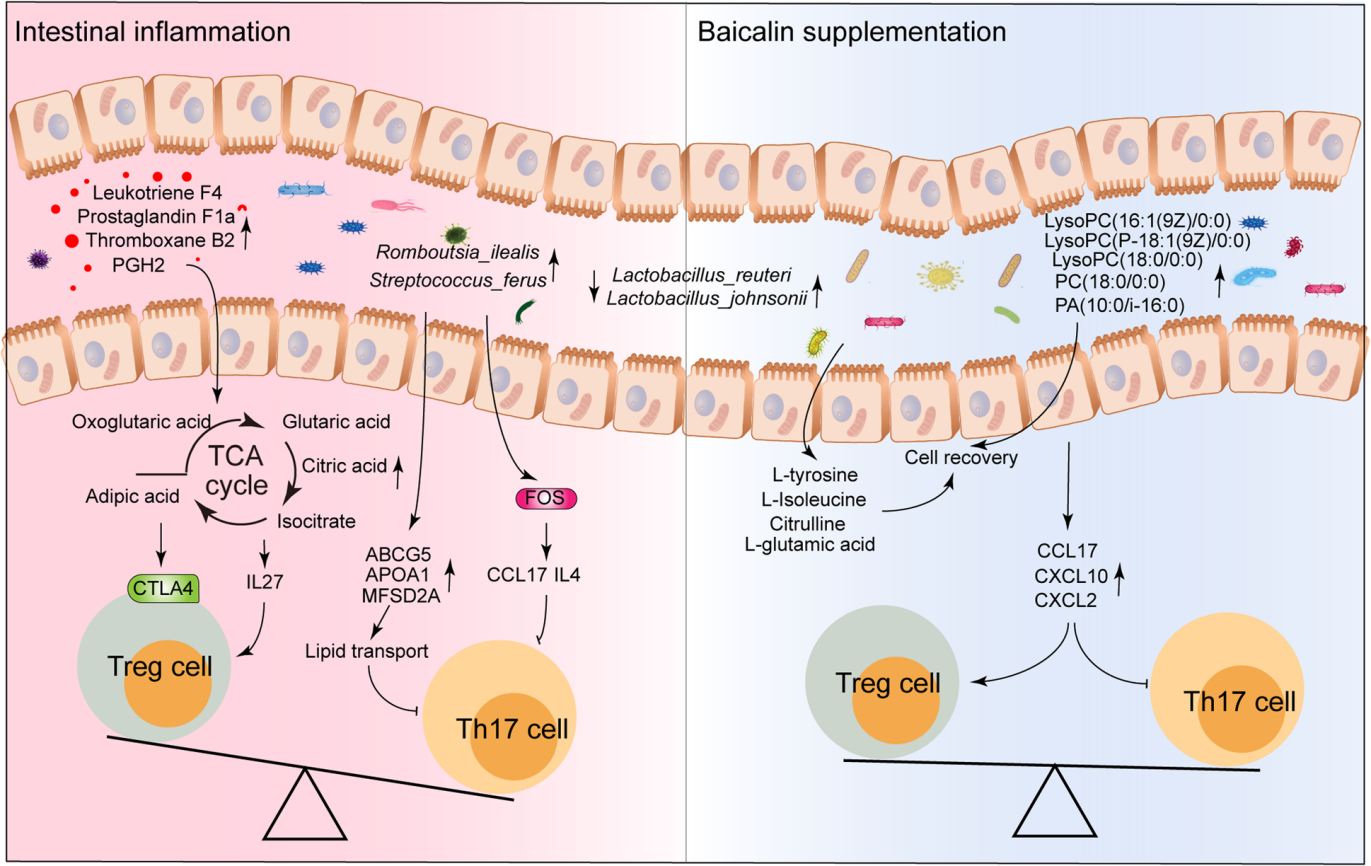

## Introduction

Intestinal inflammation is a significant health issue in piglet production, particularly during the weaning period when piglets are highly vulnerable to bacterial infections due to low immunity and weaning stress [1]. Pathogenic *Escherichia coli* (*E. coli*) is a major cause of intestinal inflammation in piglets [2], leading to intestinal damage, impaired nutrient absorption, and stunted growth [3, 4]. Chronic intestinal inflammation reduces feed efficiency and increases breeding costs [5].

Antibiotics are the primary treatment for intestinal inflammation in animal husbandry [6, 7]. However, many pathogenic *E. coli* strains have developed resistance to multiple antibiotics [8–10]. Furthermore, antibiotic treatments can disrupt the balance of intestinal microbiota and damage the intestinal barrier [11, 12]. T helper cell 17 (Th17) and regulatory T cells (Tregs) play key roles in the development and regulation of intestinal inflammation [13]. Th17 cells secrete pro-inflammatory cytokines to mediate inflammation, while Treg cells secrete anti-inflammatory cytokines to suppress the immune response and maintain immune system balance [14]. An imbalance in the Th17/Treg cell ratio is a critical pathological mechanism in many inflammatory bowel diseases (IBD) [13].

Baicalin, the main active component of the traditional Chinese medicine *Scutellaria*, is known for its anti-inflammatory, antioxidant, and antibacterial properties [15, 16]. Recent studies have confirmed that baicalin inhibits the production of bacterial DNA, RNA and proteins, and breaks down endotoxins [17, 18].

Our previous studies have also demonstrated that baicalin regulates intestinal microbiota and host immune function, thereby exerting anti-inflammatory effects and facilitating intestinal repair [11]. In vitro studies have shown that baicalin can inhibit *E. coli* in milk samples and reduce its drug resistance [17]. Additionally, baicalin has been found to inhibit lung inflammation in chickens caused by co-infection of *Mycoplasma septicus* and *E. coli* [18]. However, its role in reducing intestinal inflammation remains insufficiently explored. This study hypothesizes that baicalin supplementation can regulate immune response and microbial composition to alleviate intestinal inflammation caused by pathogenic *E. coli* in piglets. Therefore, our study aims to explore the mechanisms by which baicalin regulates intestinal health and alleviates intestinal inflammation in weaned piglets. This study offers a theoretical foundation and practical recommendations for utilizing baicalin as a nutritional intervention to enhance intestinal health in piglets.

## Materials and methods

### Piglets and experiment design

A total of 72 healthy weaned piglets of similar weight (Duroc-Landrace-Yorkshire; initial weight: 6.19 ± 0.51 kg; 24-day-old; half male and half female) were selected and assigned into 4 treatment groups with 18 piglets in each group, each consisting of 3 pens with 6 pigs per pen (1.5 m × 2.4 m). The 4 treatment groups included: 1) control group (CON), piglets were fed a basal diet; 2) *E. coli* group (*E. coli*), piglets were fed a basal diet and intraperitoneally injected with *Escherichia coli* SKLAN202302; 3) Baicalin prevention group (BL + *E. coli*), piglets were fed a basal diet containing 100 mg/kg baicalin (purity ≥ 85%, Peking Centre Technology Co., Ltd., Beijing, China) and intraperitoneally injected with *Escherichia coli* SKLAN202302; and 4) High dose baicalin prevention group (HBL + *E. coli*), piglets were fed a basal diet containing 500 mg/kg baicalin and intraperitoneally injected with *Escherichia coli* SKLAN202302. The experiment period was 18 d. On d 7, 10, and 15 of the experiment, piglets in *E. coli,* BL + *E. coli,* and HBL + *E. coli* group were intraperitoneally injected with 1 mL, 2 mL, and 3 mL of *Escherichia coli* SKLAN202302 (Concentration: 2 × 10^9^ CFU/mL), respectively. Piglets in CON group were intraperitoneally injected with equal amount of normal saline. The *Escherichia coli* SKLAN202302 strain was isolated from the colonic digesta of a diarrheal piglet in our laboratory and stored at the China General Microbiological Culture Collection Center under storage number CGMCC No.26420. Throughout the experiment, all piglets were housed indoors where the temperature was controlled at 24–26 °C and the relative humidity was maintained at 50%–70%. They were allowed free access to feed and water. The basal diets were formulated according to the National Research Council (2012) [19], as detailed in Table 1. Three days after the final injection, 8 pigs from each group were randomly selected for euthanized, and samples of the ileum and ileal chyme were collected for subsequent analysis. All experimental procedures and care protocols were reviewed and approved by the Institutional Animal Care and Use Committee (IACUC) of the Institutional Animal Care and Use Committee of the Institute of Animal Science at the Chinese Academy of Agricultural Sciences (approval number: IAS2022-113). Our study’s design, analysis, and reporting were conducted with the utmost consideration for reducing animal suffering and minimizing the number of animals used, in line with the principles of the 3Rs (Replacement, Reduction, and Refinement). The highest standards of animal welfare and ethical conduct were maintained throughout the research.

**Table 1:** Composition and nutrient level of basal diet (air-dry basis)

Item, %	Basal diet
Ingredients	
Suckling corn	28.98
Expanded corn	15.00
Broken rice	15.00
Expanded soybean	13.00
Dried whey	8.00
Soybean meal	4.50
Glucose	4.00
Fish meal	3.00
Hydrolyzed wheat protein	3.00
Soybean oil	1.50
Calcium formate	0.80
Montmorillonite	0.50
Calcium phosphate monobasic	0.50
L-Lysine hydrochloride	0.65
L-Thr	0.24
DL-Met	0.16
L-Trp	0.07
Ethoxyquin	0.10
Premix^1^	1.00
Nutrient levels^2^	
Net energy, kcal/kg	2,620
Crude protein	17.39

^1^The premix provides per kg of diet: VA: 15,000 IU; VD_3_: 4,500 IU; VE: 72.5 mg; VK_3_: 4.5 mg; VB_1_: 4.32 mg; VB_2_: 12 mg; VB_6_: 4.86 mg; VB_12_: 30 µg; Biotin: 480 µg; Folic acid: 1.764 mg; Calcium pantothenate: 19.32 mg; Nicotinamide: 41.58 mg; Cu: 110 mg; Fe: 165 mg; Zn: 80 mg; Mn: 60 mg; I: 0.8 mg; Co: 0.6 mg; Se: 0.3 mg

^2^Net energy and crude protein are calculated values

### Sample collection

The piglets were anesthetized with sodium pentobarbital solution (4%, 40 mg/kg) and euthanized for sample collection. Ileum tissue and chyme were collected, flash-frozen in liquid nitrogen, and stored in a −80 °C freezer for subsequent analysis. Fresh ileum tissue was sectioned and preserved in Carnoy’s solution (60% methanol, 30% chloroform, and 10% acetic acid) for hematoxylin and eosin (H&E) staining, and in 2.5% glutaraldehyde for preparing frozen sections to observe the ultrastructure. Fresh ileum tissue was collected and washed thoroughly with phosphate-buffered saline, and preserved in phosphate-buffered saline for flow cytometry analysis.

### Hematoxylin and eosin (H&E) and Alcian blue staining

The ileum samples of piglets were fixed in Carnoy’s solution (60% methanol, 30% chloroform, and 10% acetic acid) for more than 24 h post-collection. The fixed ileal tissues were then dehydrated and embedded in paraffin wax to prepare paraffin sections. These sections were stained with hematoxylin and eosin to observe intestinal morphology and measure villus height and crypt depth. In addition, the paraffin sections were stained with the Alcian blue-periodic acid Schiff (AB-PAS) following the manufacturer’s instruction (Solarbio, Beijing, China) to measure goblet cell numbers. The goblet cells were manually counted in 10 crypts per section. All measurements of the stained slides were performed using a DM300 light microscope (Leica, Germany).

### Ultrastructure observed by electron microscopy

The ileal tissues were initially fixed in 2.5% glutaraldehyde for 4 h at 4 °C. Following fixation, the samples underwent a series of preparation steps, including dehydration, infiltration, and embedding, culminating in the production of ultra-thin sections. These sections were then stained with lead citrate and uranyl acetate to enhance contrast. Examination was performed using a Tecnai G2 20 TWIN transmission electron microscope (FEI, Hillsboro, OR, USA). High-resolution digital images of the stained sections were captured with a GATAN ES1000W Erlangshen CCD camera.

### 16S rRNA gene sequencing analysis

Ileal chyme was collected for 16S rRNA gene sequencing analysis. Microbial DNA was extracted from the ileal chyme using the E.Z.N.A.® soil DNA Kit (Omega Biotek, Norcross, GA, USA) following the manufacturer’s protocol. The V3–V4 regions of the bacterial 16S rRNA gene were amplified using the primer pairs 338F (5′-ACTCCTACGGGAGGCAGCAG-3′) and 806R (5′-GGACTACHVGGGTWTCTAAT-3′) with an ABI Gene Amp® 9700 PCR thermocycler (ABI, CA, USA). Sequencing of the amplicon was performed on the Illumina MiSeq platform using PE300 technology, and the resulting raw reads were uploaded to NCBI (accession number: PRJNA1130863). Data pre-processing and initial analysis were conducted on the Majorbio I-Sanger cloud platform (www.i-sanger.com, Majorbio, Shanghai, China). Principal coordinates analysis (PCoA) based on weighted-UniFrac distance metrics was performed to evaluate the microbial communities among three groups. Alpha diversity was assessed using the Sobs, Shannon, and Chao indices.

### Untargeted metabolomics analyses

Ileal chyme was collected and analyzed for metabolomics using liquid chromatography-mass spectrometry (LC-MS) by Majorbio Biotech (Shanghai, China). The specific analysis methods are detailed in our previous publication [11]. The resulting raw data were preprocessed using Progenesis QI software (Waters Corporation, Milford, USA), with false positive peaks and redundancies removed. Metabolites were identified using the HMDB, Metlin (https://metlin.scripps.edu/), and Majorbio databases. Differential metabolites were identified with a VIP > 1 and *P* < 0.05. The KEGG database (http://www.genome.jp/kegg/) was used for pathway analysis and to compare metabolite concentrations.

### Transcriptome analysis

For each group, 5 ileal mucosa samples were randomly selected for total RNA extraction using the phenol–chloroform method. cDNA library construction and sequencing were performed by Majorbio Company (Shanghai, China). High-quality RNA samples were obtained through quality control measures (OD_260/280_ = 1.8–2.2, OD_260/230_ ≥ 1.0, RIN ≥ 6.5, 28S:18S ≥ 1.0, and > 10 μg). Differentially expressed gene identification, GO and KEGG functional enrichment analysis, and other processes were conducted on the Majorbio I-Sanger Cloud Platform (www.i-sanger.com), using the Benjamini–Hochberg method for *P*-value adjustment. The raw data have been submitted to the NCBI Sequence Read Archive (SRA: PRJNA1131845).

### Flow cytometry

The ileum tissue was repeatedly scraped with a cell scraper and added to 5 mL of PBS. The mixture was then filtered through a 200-mesh filter to obtain single cells. The filtrate was layered over 3 mL of lymphocyte separation solution, and lymphocytes were isolated by centrifugation. The isolated lymphocytes were washed with PBS, resuspended in 2 mL of RPMI 1640 medium, and stored at 4 °C for subsequent flow cytometry. The single-cell suspension was incubated with anti-CD3 PE (Invitrogen, #MA5-41035) and anti-CD4 PE-Cy5 (Invitrogen, #MA5-28734) for 15 min in the dark to stain the cell surface. Following incubation, a cell fixation solution was added, and the cells were fixed for 30 min in the dark, after which a cell permeabilization solution was applied. Th17 cells were analyzed by staining with anti-human IL-17A FITC (MABTECH, #3520-7). For Treg cell analysis, cells were stained with anti-Mo/RT Foxp3 PE-Cy7 (eBioscience, #25-5773-82). Stained cells were then analyzed using a FACS Canto II Flow Cytometer (BD Biosciences) with FlowJo software (FlowJo, Ashland, OR, USA).

### Western blot

Total protein was extracted from ileum mucosa and quantified using a bicinchoninic acid (BCA) protein assay kit (Thermo Fisher Scientific, MA, USA). Equal amounts of protein were loaded onto a sodium dodecyl sulfate–polyacrylamide gel for electrophoresis and then transferred onto a polyvinylidene fluoride (PVDF) membrane. The membrane was blocked with 5% skim milk for 2 h, incubated with the primary antibody overnight at 4 °C, and subsequently with an enzymic secondary antibody (Sangon Biotech, #D110058) at room temperature for 1 h. Protein signals were detected using an ECL kit (Bio-Rad, CA, USA) and visualized with the Bio-Rad Chemi XRS imaging system (Bio-Rad). The band density of the target protein was quantified and normalized to β-actin using ImageJ v1.8.0 software. The primary antibodies were sourced from the following supplier: anti-ACTB (Sangon Biotech, #D110001), Foxp3 (Beyotime, #AF6927), NLRP3 (Beyotime, #AF2155), CTLA-4 (ABclonal, #A13966), FOS (ABclonal, #A17351).

### Statistical analysis

Statistical analysis was performed using SAS 9.4 software (SAS Institute, Cary, NC, USA). Quantitative data are presented as mean ± standard deviation. One-way analysis of variance (ANOVA) was used to evaluate body weight, intestinal morphology, and WB data across groups, followed by multiple comparisons using the Least Significant Difference (LSD) method. Differences in microbial data between groups were analyzed using the Wilcoxon rank-sum test, with standardized preprocessing steps applied to the data prior to analysis. Significance was established at *P* < 0.05.

## Results

### Baicalin improved growth performance and intestinal morphology

**Fig. 1:**
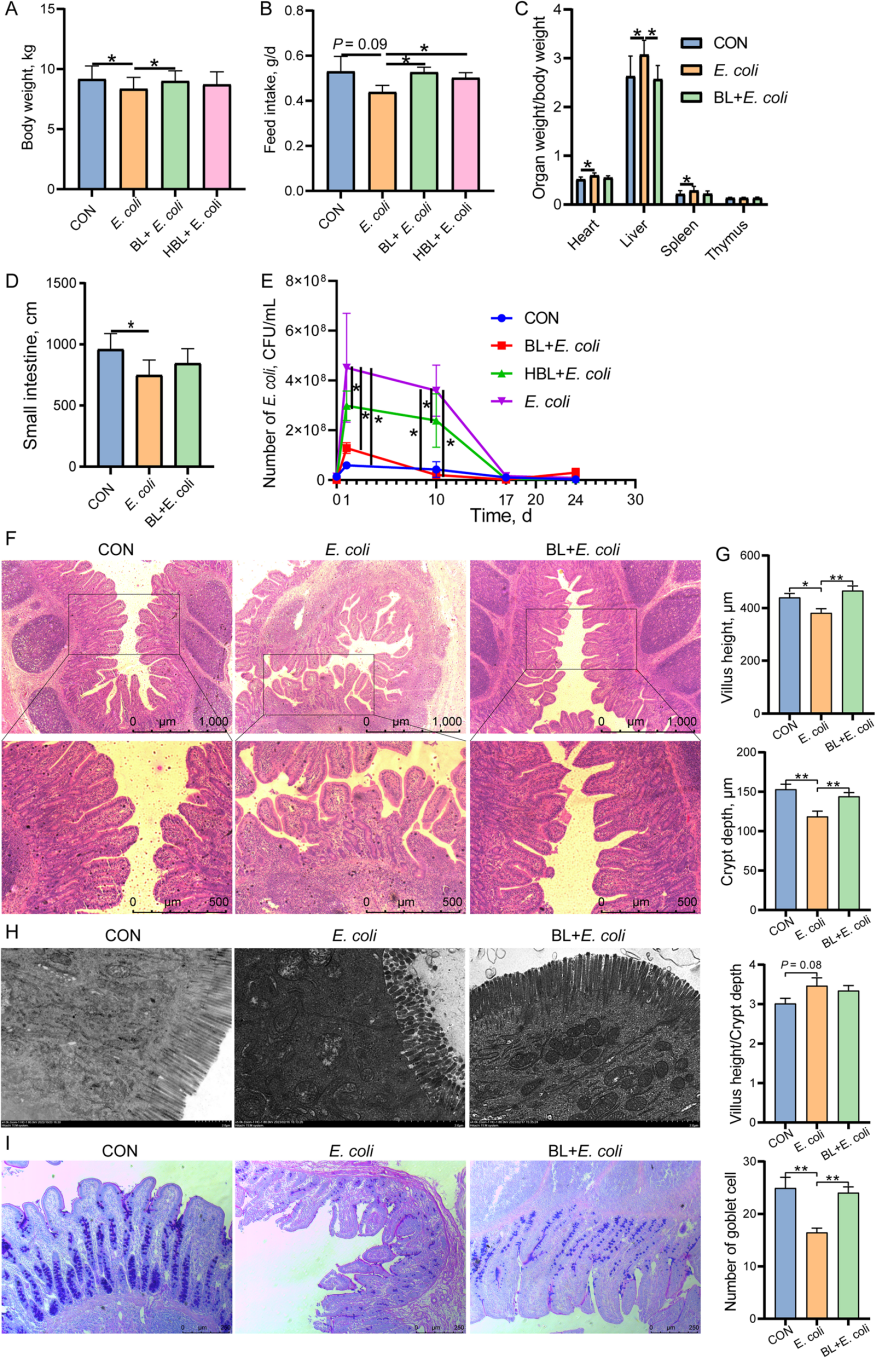
Baicalin improved growth performance and alleviated intestinal damage caused by *E. coli* infection. **A** Body weight (*n* = 18). **B** Feed intake (*n* = 3). **C** Organ weight/Body weight (*n* = 8). **D** Small intestine length (*n* = 8). **E** Number of the *E. coli* in piglet feces (*n* = 8). **F** Hematoxylin and eosin (H&E) sections of ileum. Scale bar: 500 μm and 1,000 μm. **G** Villus height, crypt depth, and villus height/crypt depth of ileum (*n* = 8). **H** Mitochondrial morphology and structure in apical enterocytes of the ileum (Scale bars, 2 µm). **I** Representative PAS staining of ileum tissue sections and the number of goblet cells (*n* = 8). Scale bar: 250 μm. Data are expressed as the mean ± SD and one-way ANOVA was performed, followed by LSD test. * means *P* < 0.05, ** means *P* < 0.01

We evaluated the effect of baicalin on growth performance and intestinal morphology in piglets challenged with *E. coli*. Baicalin supplementation improved the body weight and feed intake of the piglets compared to the *E. coli* group (*P* < 0.05), with the low dose of baicalin showing greater improvement than the high dose (Fig. 1A and B). Additionally, baicalin significantly reduced the number of *E. coli* in piglet feces, with the BL + *E. coli* group having lower *E. coli* counts than the HBL + *E. coli* group (*P* < 0.05, Fig. 1E). Based on these results, piglets in the BL + *E. coli* group were selected for subsequent analysis instead of those in the HBL + *E. coli* group. *E. coli* infection caused an increase in the proportion of the heart, liver, and spleen, and a reduction in small intestine length (*P* < 0.05, Fig. 1C and D), which were ameliorated by baicalin supplementation. Baicalin also alleviated the morphological damage to the ileum caused by *E. coli*, as evidenced by increased villus height and crypt depth compared to the *E. coli* group (Fig. 1F and G). Further TEM ultrastructural analysis showed that the ileum of piglets in the CON and BL + *E. coli* groups displayed parallel and well-developed microvilli covering the apical surface of epithelial cells, with well-preserved normal mitochondrial structures (Fig. 1H). However, *E. coli* infection resulted in sparse, short, and irregular microvilli in the ileum and led to a preferential accumulation of degenerating and damaged mitochondria in ileal epithelial cells, with some cases showing complete matrix disappearance and fragmented cristae (Fig. 1H). Baicalin supplementation improved the microvilli morphology and mitochondrial structure of the ileum following *E. coli* infection (Fig. 1H). The number of goblet cells in the ileum significantly decreased after *E. coli* challenge, while baicalin supplementation significantly increased the number of goblet cells (*P* < 0.05, Fig. 1I).

### Baicalin regulated the ileal microbiota composition

**Fig. 2:**
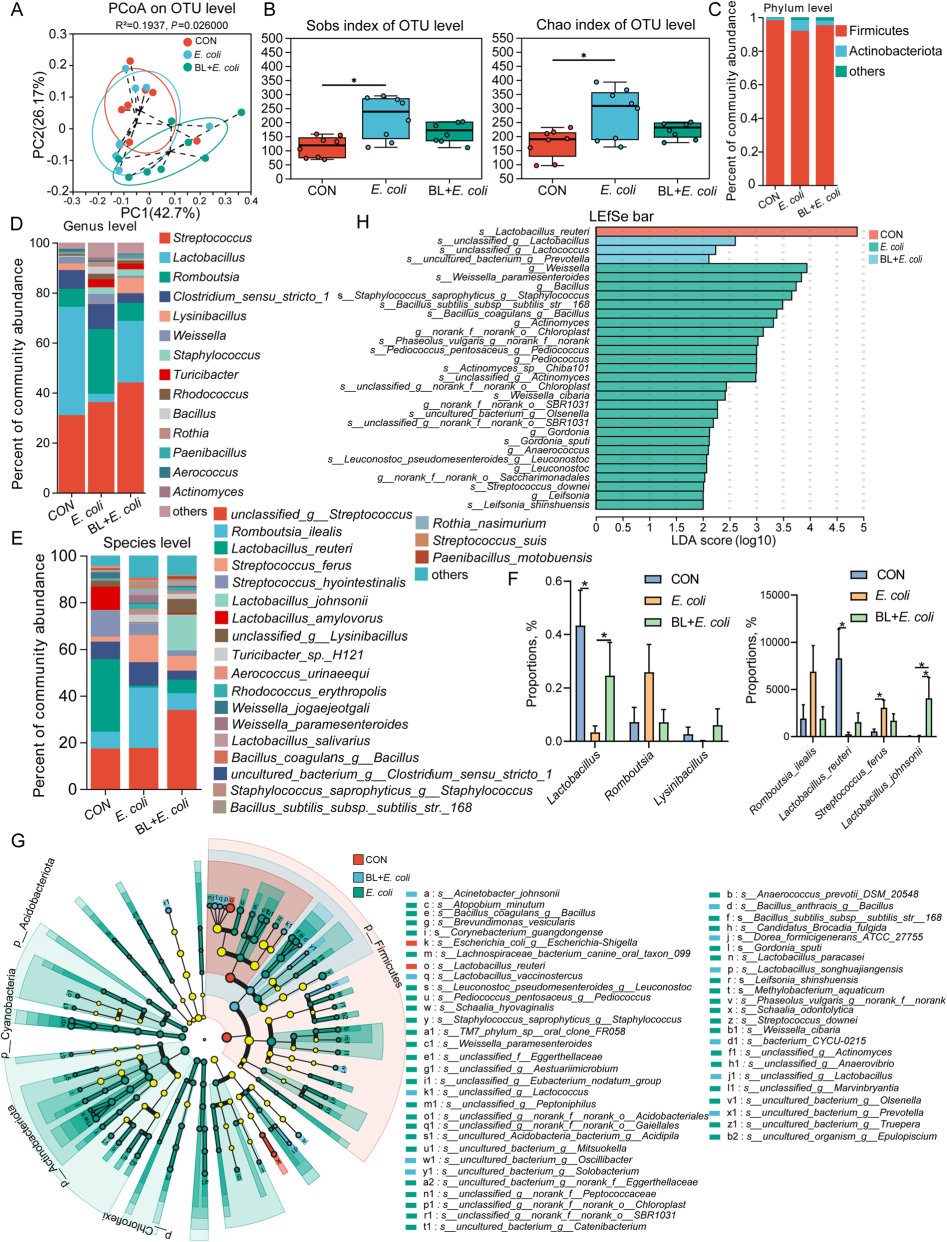
Baicalin regulated the ileal microbiota composition disrupted by *E. coli* infection (*n* = 8). **A** PCoA analysis of microbiota in ileum based on weighted UniFrac distance metrics. **B** Alpha-diversity (Sobs and Chao index) of microbiota. Intergroup difference test was performed by Wilcoxon rank-sum test. * means *P* < 0.05. **C–E** Relative abundance of microbiota at phylum level (**C**), genus levels (**D**), and species level (**E**). Less than 1% abundance of phyla or genus was merged into others. **F** Proportions of key genus and species in three groups. Intergroup difference test was performed by Wilcoxon rank-sum test. * means *P* < 0.05. **G** LEfSe analysis of microbiota. **H** LEfSe Bar

The microbiota composition in the ileal chyme was analyzed using 16S rDNA sequencing. PCoA analysis based on weighted UniFrac revealed marked distinctions between the *E. coli* group and both the CON and BL + *E. coli* groups, with significant differences confirmed by ANOSIM analysis (Fig. 2A, *P* < 0.05). Alpha-diversity indices, including Sobs, Chao, and Shannon, were higher in the *E. coli* group than in the CON group (Fig. 2B, *P* < 0.05). The microbiota composition was evaluated and presented in Fig. 2C–E. At the phylum level, the proportion of Actinobacteriota was higher, while Firmicutes was lower in the *E. coli* group compared to the CON and BL + *E. coli* groups (Fig. 2C). At the genus level, *Lactobacillus* and *Lysinibacillus* were notably diminished in the *E. coli* group, whereas *Romboutsia*, *Weissella*, *Turicibacter*, *Bacillus*, and *Rothia* were relatively abundant (Fig. 2D). The microbial composition of the BL + *E. coli* group was more similar to that of the control group. The abundance of *Lactobacillus* in the *E. coli* group was significantly lower than in the CON and BL + *E. coli* groups (*P* < 0.05, Fig. 2F). At the species level, *Lactobacillus_reuteri*, *Lactobacillus_amylovorus*, and *unclassified_g__Lysinibacillus* were notably diminished in the *E. coli* group, while *Romboutsia_ilealis*, *Streptococcus_ferus*, *Weissella_paramesenteroides*, and *Rothia_nasimurium* were relatively abundant (Fig. 2E). These proportions recovered after baicalin supplementation. Interestingly, the abundance of *Lactobacillus_reuteri* was significantly lower, while the abundance of *Streptococcus_ferus* was significantly higher in the *E. coli* group (*P* < 0.05, Fig. 2F). Supplementation with baicalin significantly increased the abundance of *Lactobacillus_johnii* (*P* < 0.05, Fig. 2F). Dissimilarities in the ileal microbiota were identified using the Linear Discriminant Analysis Effect Size (LEfSe) method (Fig. 2G and H). The most significantly represented species in the CON group was *Lactobacillus_reuteri*, while in the BL + *E. coli* group, *unclassified_g__Lactobacillus* and *unclassified_g__Lactococcus* were predominant. In contrast, *Weissella*, *Weissella_paramesenteroides*, *Bacillus*, and *Staphylococcus_saprophyticus_g__Staphylococcus* were the most represented in the *E. coli* group (Fig. 2H).

### Baicalin regulated ileal metabolism

**Fig. 3:**
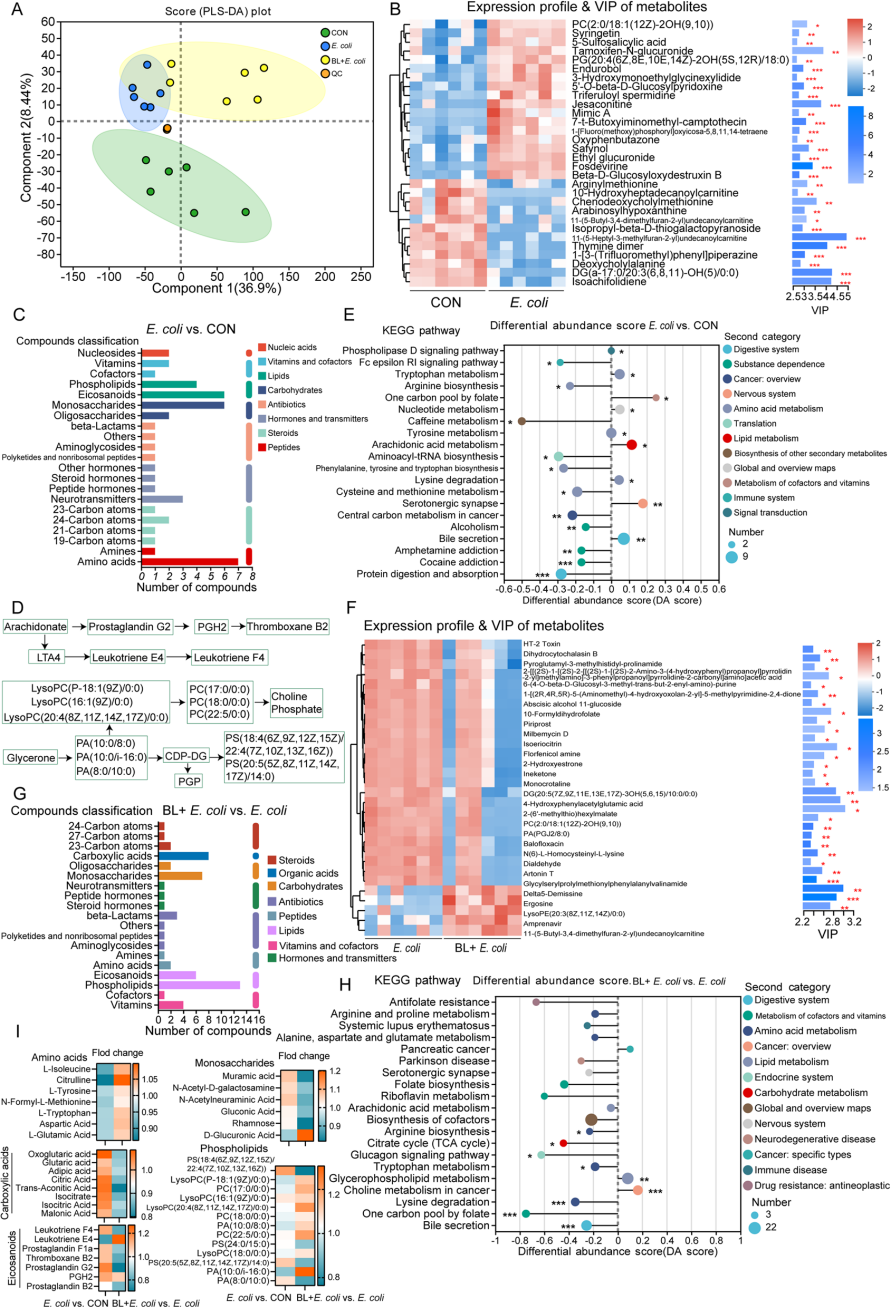
Baicalin regulated ileal metabolism disrupted by *E. coli* infection (*n* = 8). **A** PLS-DA analysis. **B** Expression profile and VIP analysis of metabolites for CON and *E. coli* group. **C** Compounds classification analysis of differential metabolites for *E. coli* vs. CON group. **D** Schematic diagram of metabolism and transformation processes of eicosanoids and glycerol phospholipid. **E** KEGG pathway enrichment topology analysis for differential metabolites of *E. coli* vs. CON group. **F** Expression profile and VIP analysis of metabolites for BL + *E. coli* and *E. coli* group. **G** Compounds classification analysis of differential metabolites for differential metabolites of BL + *E. coli* vs. *E. coli* group. **H** KEGG pathway enrichment topology analysis for differential metabolites of BL + *E. coli* vs. *E. coli* group. **I** Fold change of amino acids, carboxylic acids, eicosanoids, monosaccharides, and phospholipids for *E. coli* vs. CON group and BL + *E. coli* vs. *E. coli* group. * means *P* < 0.05, ** means *P* < 0.01, *** means *P* < 0.001

Untargeted metabolomic profiling of ileal chyme was conducted to evaluate the influence of baicalin on metabolism. Principal component analysis (PCA) revealed distinct differences among the three groups (Fig. 3A). In the differential metabolites between the *E. coli* and CON groups, PC [2:0/18:1 (12Z)−2OH (9,10)], syringetin, 5-sulfosalicylic acid, and tamoxifen-N-glucuronide were significantly up-regulated, while arginylmethionine, 10-hydroxyheptadecanoylcarnitine, chenodeoxycholylmethionine, and arabinosylhypoxanthine were significantly down-regulated. These metabolites had the most substantial influence on the observed differences (Fig. 3B). Many of the differentially enriched metabolites in the *E. coli* group were amino acids, monosaccharides, eicosanoids, and phospholipids (Fig. 3C). Notably, metabolites of eicosanoids, including leukotriene F4, prostaglandin F1a, leukotriene E4, thromboxane B2, prostaglandin G2, and PGH2, were significantly increased in the *E. coli* group (Fig. 3D and I). These metabolites are known inflammatory mediators that exacerbate the inflammatory response. KEGG pathway analysis based on differential metabolites highlighted the top pathways, including the phospholipase D signaling pathway, Fc epsilon RI signaling pathway, tryptophan metabolism, and arginine biosynthesis. In the BL + *E. coli* vs. *E. coli* groups, HT-2 toxin, dihydrocytochalasin B, and pyroglutamyl-3-methylhistidyl-prolinamide were significantly down-regulated, while delta5-Demissine, ergosine, lysoPE [20:3(8Z,11Z,14Z)/0:0], and imipramine were significantly up-regulated (Fig. 3F). Many of the differentially enriched metabolites in the BL + *E. coli* group were phospholipids, eicosanoids, monosaccharides, and carboxylic acids (Fig. 3G). Specifically, metabolites of glycerol phospholipid, including lysoPC [P-18:1(9Z)/0:0], PC (17:0/0:0), lysoPC [16:1(9Z)/0:0], PC(18:0/0:0), lysoPC (18:0/0:0), PA (10:0/i-16:0), and PA (10:0/8:0), were significantly decreased in the *E. coli* group but increased in the BL + *E. coli* group, indicating their role in the regulation of inflammatory response (Fig. 3D and I). KEGG pathway analysis of differential metabolites in the BL + *E. coli* vs. *E. coli* groups identified the top pathways, including antifolate resistance, arginine and proline metabolism, systemic lupus erythematosus, alanine, aspartate and glutamate metabolism, and pancreatic cancer (Fig. 3H). Among the differential metabolites, carboxylic acids, eicosanoids, monosaccharides, and phospholipids were increased, while amino acids were decreased in the *E. coli* vs. CON groups. However, baicalin supplementation produced opposite effects on these metabolites compared to *E. coli* alone (Fig. 3I).

### Baicalin regulated transcriptome

**Fig. 4:**
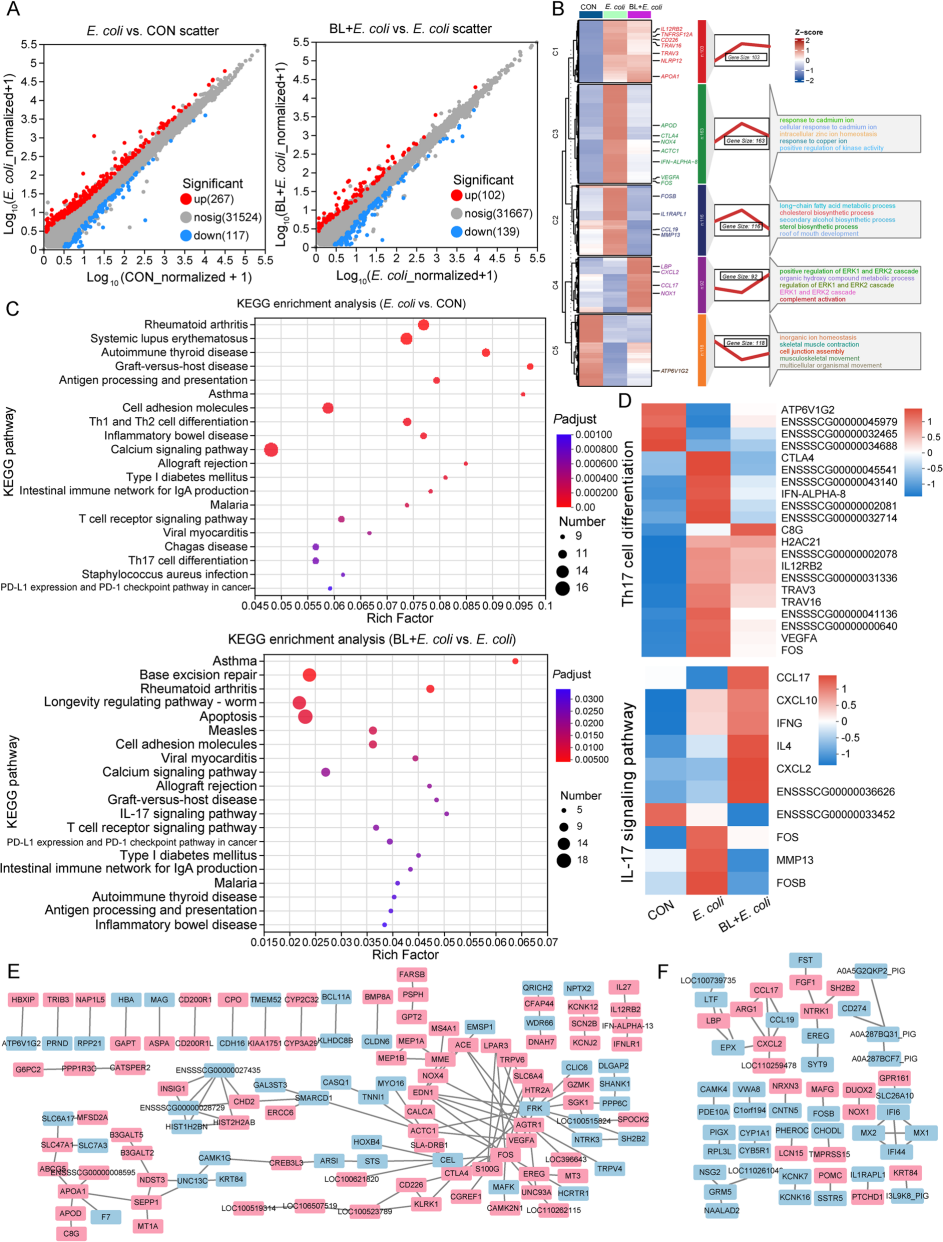
Baicalin regulated gene expression caused by *E. coli* infection (*n* = 5). **A** Scatter plot of expression difference (Fold change > 1.8, *P* < 0.05). Each dot represents an individual gene. Red dots represent the significantly up-regulated genes, blue dots represent the significantly down-regulated genes, and gray dots represent non-significant differential genes. **B** Clustering heat map, trend analysis and enriched GO terms for all differential genes in *E. coli* vs. CON and BL + *E. coli* vs. *E. coli* group. **C** KEGG enrichment analysis of differential genes in *E. coli* vs. CON and BL + *E. coli* vs. *E. coli* group. **D** Th17 cell differentiation pathways and IL-17 signaling pathway gene expression heatmap. **E** and **F** The main network cluster of genes different expression between *E. coli* and CON (**E**), and between BL + *E. coli* and *E. coli* (**F**). Each nodes representing a gene, and edges each representing an interaction between two genes. Blue nodes represent genes that are significantly up-regulated, and pink nodes represent genes that are significantly down-regulated

Transcriptome analysis of ileum tissue was conducted to further explore the effect of baicalin on *E. coli*-infected piglets. A total of 384 differential expression genes (DEGs) were identified in the *E. coli* vs. CON group, with 267 up-regulated and 117 down-regulated (Fold change > 1.8, *P* < *0.05*, Fig. 4A). GO enrichment analysis revealed that the up-regulated DEGs were mainly involved in the response to cadmium ion, intracellular zinc ion homeostasis, long-chain fatty acid metabolic process, and cholesterol biosynthetic process (Fig. 4B). In contrast, the down-regulated DEGs were involved in inorganic ion homeostasis, skeletal muscle contraction, and cell junction assembly. KEGG enrichment analysis showed that these DEGs were primarily involved in autoimmune disease and inflammatory pathways, including rheumatoid arthritis (RA), systemic lupus erythematosus (SLE), autoimmune thyroid disease (AITD), graft-versus-host disease (GD), intestinal immune network for IgA production, inflammatory bowel disease, Th1 and Th2 cell differentiation, and Th17 cell differentiation. Autoimmune diseases such as multiple sclerosis (MS) and Alzheimer’s disease (AD) are closely associated with Th17 cell differentiation. Genes related to these autoimmune diseases and Th17 cell differentiation, including *CTLA4*, *IFN-ALPHA-8*, *IL12RB2*, *TRAV3*, *TRAV16*, *FOS*, and *VEGFA*, were up-regulated in the *E. coli* group but down-regulated in the BL + *E. coli* group (*P* < 0.05, Fig. 4D). In addition, genes involved in lipid metabolism (*SLC47A1*, *MFSD2A*, *ABCG5*, *APOA1*, *APOD*, *C8G*), glucose metabolism (*B3GALT5*, *B3GALT2*, *NDST3*, *SEPP1*, *MT1A*), and inflammatory reaction (*IL27*, *IL12RB2*, *IFN-ALPHA-13*, *IFNLR1*, *EREG*, *CD226*), which are closely linked to Th17 cell differentiation, were significantly up-regulated following *E. coli* infection (Fig. 4E). Protein network analysis indicated that genes such as *FOS*, *CTLA4*, *APOA1*, *CEL*, *FRK*, and *AGTR1* had the strongest association with other genes, highlighting their crucial roles in *E. coli* infection (Fig. 4E).

Baicalin supplementation significantly restored gene expression changes caused by *E. coli* infection, with the gene expression trends in the BL + *E. coli* group more closely resembling the control group and contrasting with the *E. coli* group (Fig. 4B). GO enrichment analysis showed that baicalin may enhance signaling pathways associated with cell survival and proliferation (positive regulation of the ERK1 and ERK2 cascade), metabolic processes (organic hydroxy compound metabolic process), and immune responses (complement activation), contributing to the restoration of normal cellular functions following *E. coli* infection (Fig. 4B). KEGG enrichment analysis indicated that DEGs in the BL + *E. coli* vs. *E. coli* group were primarily involved in base excision repair, rheumatoid arthritis, longevity regulating pathway–worm, apoptosis, IL-17 signaling pathway, and T cell receptor signaling pathway (Fig. 4A and C). DEGs related to the IL-17 signaling pathway, including *CCL17*, *CXCL10*, *IFNG*, *IL4*, and *CXCL2*, were up-regulated in the BL + *E. coli* group compared to the *E. coli* group, whereas *FOS*, *FOSB*, and *MMP13* were down-regulated (Fig. 4D). Protein network analysis revealed that genes such as *CXCL2*, *CCL17*, *LBP*, *ARG1*, *NTRK1*, and *FGF1* had the strongest associations with other genes, suggesting their significant roles in baicalin supplementation (Fig. 4F).

### Correlation analysis between microbiota and metabolome

**Fig. 5:**
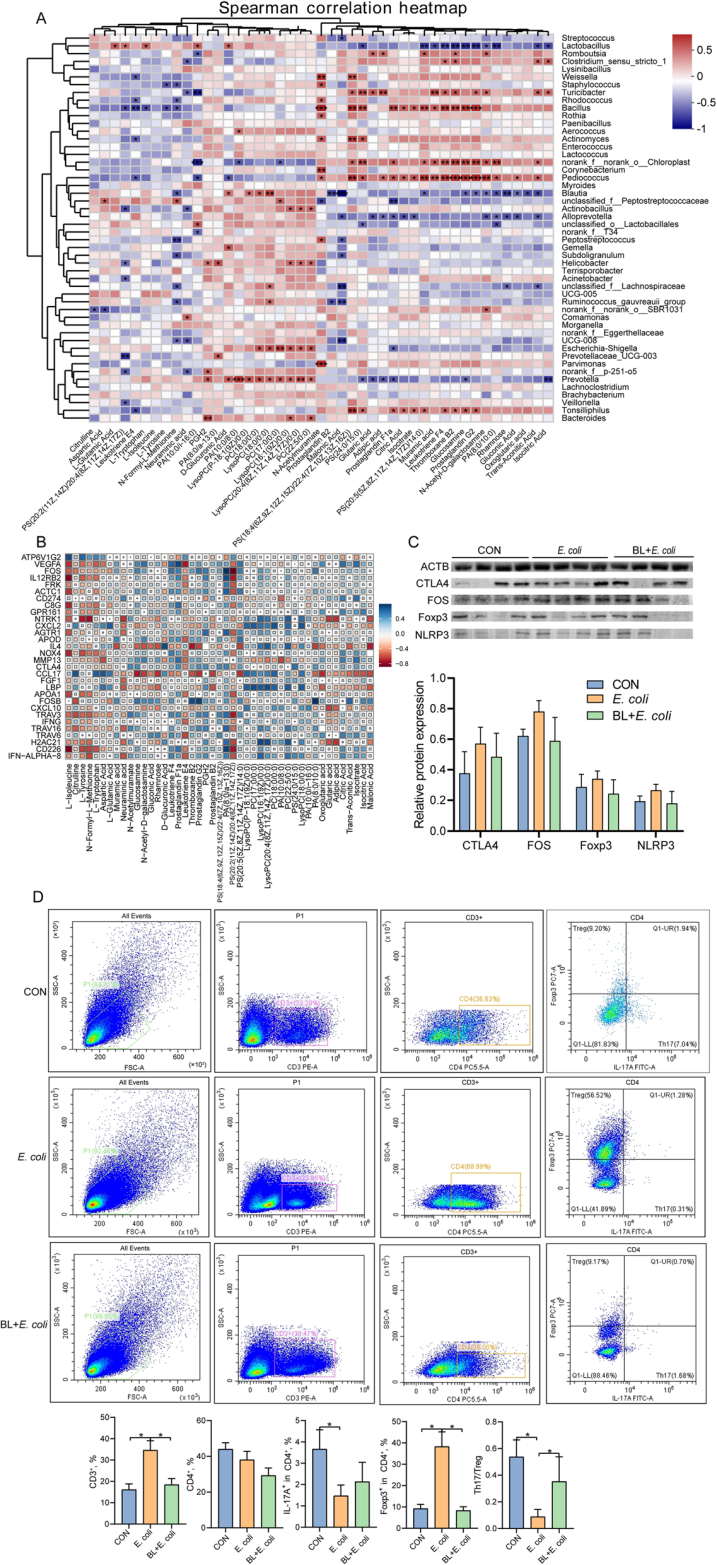
Association analysis. **A** Spearman’s correlation between gut microbiota and differential metabolites at genus level. Color gradient denoting Spearman’s correlation coefficients. The correlation was assessed at the genus level by partial (geographic distance–corrected) Mantel tests. Edge width corresponds to Mantel’s r statistic for the corresponding distance correlations, and edge color denotes the statistical significance based on 9,999 permutations. **B** Spearman’s correlation between metabolome and transcriptome. **C** Western blotting analysis of expression of CTLA4, FOS, Foxp3, NLRP3, and ACTB in the ileum, and the ratio of cleaved form to full from of CTLA4, FOS, Foxp3, and NLRP3 (*n* = 4). Intergroup difference test was performed by one-way ANOVA. **D** Flow cytometry analysis of ileum tissue (*n* = 8). Data are expressed as the mean ± SD and one-way ANOVA was performed, followed by LSD test. * means *P* < 0.05, ** means *P* < 0.01

A Spearman correlation analysis was conducted on the differential metabolites of amino acids, monosaccharides, eicosanoids, and phospholipids with the top 50 genera to determine the relationship between potential metabolites and major microbiota (Fig. 5A). The results indicated that phospholipids were positively correlated with *Blautia*, *unclassified_f__Peptostreptococcaceae*, *Actinobacillus*, *Helicobacter*, *Escherichia-Shigella*, *Prevotella*, and *Bacteroides*. Metabolites such as glutaric acid, adipic acid, prostaglandin F1a, citric acid, isocitrate, muramic acid leukotriene F4, thromboxane B2, glucosamine, prostaglandin G2, and N-Acetyl-D-galactosamine showed positive correlations with *Romboutsia*, *Clostridium_sensu_stricto_1*, *Weissella*, *Turicibacter*, *norank_f__norank_o__Chloroplast*, and *Pediococcus*, while displaying negative correlations with *Lactobacillus*, *Blautia*, *Alloprevotella*, and *Prevotella*.

### Correlation analysis between metabolome and transcriptome

The relationship between the differential metabolism of amino acids, monosaccharides, eicosanoids, and phospholipids and potential DEGs is illustrated in Fig. 5B. Amino acids were positively correlated with *VEGFA*, *FOS*, *IL12RB2*, *FRK*, *ACTC1*, *NTRK1*, *NOX4*, *APOA1*, *CTLA4*, *TRAV3*, and *CD226*, and negatively correlated with *IL-4*, *ATP6V1G2*, *CCL17*, and *LBP*. Eicosanoids showed positive correlations with *IL-4*, *ATP6V1G2*, *CCL17*, and *LBP*, while exhibiting negative correlations with *VEGFA*, *FOS*, *IL12RB2*, *FRK*, *ACTC1*, *NTRK1*, *NOX4*, *APOA1*, *CTLA4*, *TRAV3*, and *CD226*. Phospholipids were negatively correlated with these DEGs. Carboxylic acids were positively correlated with *NTRK1*, *CXCL2*, *IL4*, *CCL17*, *CXCL10*, and *H2AC21*.

### Flow cytometry and Western blot analysis

To validate the transcriptome results, the expression levels of *FOS*, *CTLA4*, *Foxp3*, and *NLRP3*, which are involved in Th17 cell differentiation, were assessed using Western blot analysis. The results indicated that the protein expression patterns were consistent with those obtained from the transcriptome analysis (Fig. 5C). To further determine the effect of baicalin on Th17 cell differentiation in piglets infected with *E. coli*, flow cytometry was employed to analyze the number and proportion of Th17 and Treg cells in the ileum (Fig. 5D). The results demonstrated that baicalin supplementation counteracted the increase in the proportion of CD3^+^ cells and Foxp3^+^ cells caused by *E. coli* infection. Additionally, baicalin reversed the decrease in the proportion of IL-17A^+^ cells and the Th17/Treg ratio caused by *E. coli* infection.

## Discussion

Intestinal inflammation is a common and serious issue in piglet production, leading to reduced feed efficiency, impaired growth, and economic losses. *E. coli* is a primary pathogen causing intestinal inflammation in piglets. In this study, *E. coli* was isolated from piglets with diarrhea, resulting in decreased body weight and feed intake, intestinal morphological changes, a reduction in goblet cells, and other signs of intestinal damage. These findings align with previously reported phenotypes in piglets infected with pathogenic *E. coli* [20]. However, baicalin supplementation significantly reduced the fecal *E. coli* content of piglets, restored body weight and feed intake, and improved intestinal villus structure and goblet cell count.

*E. coli* infection may affect the colonization of symbiotic bacteria by competing for nutrients and adhesion sites or through toxin release [21]. In this study, *E. coli* infection significantly reduced the abundance of *Lactobacillus* and *Lysinibacillus*, particularly *Lactobacillus_reuteri* and *Lactobacillus_amylovorus*. Conversely, the abundance of *Romboutsia*, *Bacillus*, and *Rothia* increased following *E. coli* infection, including *Romboutsia_ilealis*, *Streptococcus_ferus*, *Weissella_paramesenteroides*, and *Rothia_nasimurium*. Interestingly, baicalin supplementation restored the abundance of these symbiotic bacteria. *Rothia_nasimurium* has been reported to cross immune barriers, posing a risk of illness and death to birds or other animals [22]. *Lactobacillus_reuteri,* a dominant intestinal bacterium in piglets [23], is known to enhance host immunity and alleviate intestinal inflammation [24–26]. *Lactobacillus_amylosus* has various probiotic functions, including protecting the intestinal barrier, inhibiting pathogen colonization, and reducing inflammation [27–29].

In this study, we observed the effects of *E. coli* infection on metabolite profiles in piglets and explored the regulatory role of baicalin supplementation on these changes. The results showed that *E. coli* infection significantly increased the levels of several metabolites, including oxoglutaric acid, glutaric acid, adipic acid, citric acid, isocitrate, *trans*-aconitic acid, malonic acid and other intermediates of the TCA cycle [30–32]. This suggests that the infection leads to a significant enhancement in energy metabolism, possibly due to increased metabolic demands on cells in response to pathogen invasion [32, 33]. Concurrently, the elevation of eicosanoids such as leukotriene F4, prostaglandin F1a, thromboxane B2, and PGH2 indicates the activation of a strong inflammatory and immune response [34, 35]. These inflammatory mediators are released in large quantities during bacterial infection, promoting an inflammatory response to defend against pathogens [36, 37]. Recent studies have found that the intestinal microenvironment is rich in prostaglandin E2, which can drive mitochondrial depolarization of T cells and enhance glutathione synthesis to clear reactive oxygen species (ROS) produced by mitochondrial depolarization [38]. Eicosanoids can directly affect inflammatory T cell development, causing juvenile T cells to differentiate into pro-inflammatory Th17 and Th1 cells [39–41], while antagonistic eicosanoids reduce the severity of Th17 and Th1 cell-mediated inflammation and colitis [42]. Elevated levels of eicosanoid metabolites have been observed in the gastrointestinal mucosa of patients with IBD [43, 44]. In addition, increases in muramic acid, N-Acetyl-D-galactosamine, N-acetylneuraminic acid, gluconic acid, and rhamnose may be related to the remodeling of cell wall components and changes in glucose metabolism, common responses to infection [45]. Remarkably, baicalin supplementation significantly reduced the levels of these metabolites, suggesting that baicalin may have anti-inflammatory and metabolic regulatory effects. Furthermore, increased levels of phospholipids and lysophospholipids were observed after baicalin supplementation, indicating the promotion of membrane repair and remodeling processes [46, 47]. Phospholipids are crucial components of cell membranes, and their increased levels may reflect the process by which cells undergo repair after inflammation and infection [48]. The increase in amino acids such as L-isoleucine, citrulline, L-tyrosine, L-tryptophan, aspartic acid, L-glutamic acid may be related to protein synthesis, cell repair, and immune response [49, 50]. The elevated levels of these amino acids further support the role of baicalin in promoting cell recovery and metabolic balance after infection. Correlation analysis revealed that the changes of these metabolites were significantly correlated with the abundance of genus-level microbiota such as *Lactobacillus*, *Bacillus*, *Pediococcus*, *Blautia*, and *Tonsilliphilus*.

The results of transcriptomic analysis further revealed the extensive effects of *E. coli* infection on gene expression. DEGs were primarily concentrated in pathways related to autoimmune disease, inflammatory bowel disease, and Th17 cell differentiation. Studies have confirmed that the balance between Th17 and Treg cells is a critical mechanism affecting the occurrence and progression of autoimmune diseases and IBD [13, 51, 52]. Th17 cells, a subgroup of T helper cells characterized by IL-17 secretion [53], play a key role in combating bacterial and fungal infections. However, in autoimmune diseases, overactive Th17 cells can lead to tissue damage and chronic inflammation [54, 55]. Conversely, Treg cells, characterized by the expression of Foxp3 transcription factor, primarily function to suppress immune response and maintain immune tolerance [56]. In this study, *E. coli* infection up-regulated key genes in the Th17 cell differentiation pathway, including *FOS*, *VEGFA*, *IL12RB2*, *IFN-ALPHA-8*, and *IFN-ALPHA-13,* indicating widespread activation of the immune system. This activation was further confirmed by an increase in the number of T cells, which recruit and proliferate in response to infection [57]. During the later stages of infection, to prevent excessive inflammation and tissue damage, the immune system up-regulated immunosuppressive genes such as *CTLA4* and *IL27*, promoting the differentiation and function of Treg cells and inhibiting excessive Th17 cell response [58–60]. This was corroborated by flow cytometry, which showed an increase in Treg cells and a decrease in Th17 cells. In addition, the up-regulation of genes related to lipid transport and glucose metabolism demonstrated that *E. coli* infection affected Th17 cell differentiation at the metabolic level [61]. For instance, *MFSD2A,* involved in the transport of fatty acids such as docosahexaenoic acid (DHA), influences Th17 cell differentiation and function by regulating fatty acids availability [62]. *ABCG5,* a cholesterol transporter, is involved in the excretion of cholesterol and phytosterols [63], with high cholesterol levels promoting Th17 cell differentiation. *ABCG5* indirectly affects Th17 cell differentiation by regulating cholesterol levels [64]. High-density lipoprotein (HDL) inhibits Th17 cell differentiation and promotes Treg cell differentiation, with *APOA1* being a major protein component of HDL [65]. Interestingly, these genes were significantly down-regulated following baicalin supplementation, suggesting that excessive inflammation and immune responses could be modulated. Baicalin supplementation also upregulated the expression of genes involved in the IL-17 signaling pathway, including *CCL17*, *CXCL10*, *IFNG*, *IL4*, and *CXCL2*. Chemokines *CCL17*, *CXCL10*, and *CXCL2* recruit Treg cells to sites of inflammation and inhibit Th17 cells differentiation [66]. *IFNG* and *IL4* inhibit Th17 cell differentiation by blocking the expression of *RORγt*, a key transcription factor of Th17 cells [67]. This indicates that baicalin may promote immune regulation and apoptosis by modulating these signaling pathways, thereby alleviating the pathological changes caused by *E. coli* infection. After baicalin treatment, the proportions of T cells, Treg cells, and Th17 cells returned to control levels, indicating that baicalin can restore immune homeostasis and correct immune imbalance caused by infection. In summary, these results suggest that *E. coli* infection result in a complex immunomodulatory response, promoting the increase of Treg cells to suppress excessive inflammatory responses. Baicalin supplementation helps to restore immune balance, reduce inflammation, and regulate the ratio of Th17/Treg cells.

## Conclusions

*E. coli* infection alters gut microbiota composition, significantly reprograms metabolism, and triggers immune responses. This leads to enhanced TCA cycle activity, elevated inflammatory mediators, and an imbalance in immune cell proportions. Baicalin supplementation regulated intestinal microbiota composition and metabolic pathways, inhibited inflammatory mediator production, and restored immune cell balance, demonstrating notable anti-inflammatory and immunomodulatory effects. These findings provide a scientific basis for using baicalin as a potential therapeutic agent in animal health management and disease treatment.

## Data Availability

The original contributions presented in the study are included in the article, and further inquiries can be directed to the corresponding author. All raw sequences are deposited in the SRA Bioproject: PRJNA1131845 and PRJNA1130863.

## Abbreviations

AB-PAS: Alcian blue-periodic acid Schiff
AD: Alzheimer’s disease
ANOVA: Analysis of variance
AITD: Autoimmune thyroid disease
DEGs: Differential expression genes
DHA: Docosahexaenoic acid
*E. coli*: *Escherichia coli*
GD: Graft-versus-host disease
H&E: Hematoxylin and eosin
HDL: High-density lipoprotein
IBD: Inflammatory bowel disease
LC-MS: Liquid chromatography-mass spectrometry
LEfse: Linear discriminant analysis effect size
LSD: Least significant difference
LysoPC: Lyso phosphatidylcholine
MS: Multiple sclerosis
PC: Phosphatidylcholine
PCA: Principal component analysis
PCoA: Principal coordinates analysis
PGH2: Prostaglandin H2
RA: Rheumatoid arthritis
ROS: Reactive oxygen species
SLE: Systemic lupus erythematosus
TCA: Tricarboxylic acid
Th17: T helper cell 17
Treg: Regulatory T cells
